# Farnesoid X Receptor Attenuates the Tumorigenicity of Liver Cancer Stem Cells by Inhibiting STAT3 Phosphorylation

**DOI:** 10.3390/ijms26031122

**Published:** 2025-01-28

**Authors:** Wenling Ye, Yang Zhao, Yibo Wang, Yahan Wang, Huan Zhang, Fengling Wang, Weidong Chen

**Affiliations:** 1 Key Laboratory of Receptors-Mediated Gene Regulation and Drug Discovery, School of Basic Medical Science, Inner Mongolia Medical University, Hohhot 010110, China; 2 Key Laboratory of Receptors-Mediated Gene Regulation and Drug Discovery, School of Basic Medical Sciences, Henan University, Kaifeng 475001, China

**Keywords:** hepatocellular carcinoma, CSCs, FXR, STAT3, SOCS3

## Abstract

The Farnesoid X receptor (FXR) has recently been identified as being closely associated with the progression of primary hepatocellular carcinoma. Cancer stem cells (CSCs) play a crucial role in tumor initiation, progression, invasion, metastasis, recurrence, and drug resistance. The elucidation of the role and regulatory mechanism of FXR in CSCs is therefore deemed significant. Here, bioinformatics analysis has revealed a downregulation of FXR in hepatocellular carcinoma (HCC), which showed a negative correlation with HCC malignancy. This result was further confirmed through clinical sample analysis. Subsequently, CSCs were isolated from HCC cell lines and exhibited a significant decrease in the expression of FXR. The activation of FXR resulted in a remarkable inhibition of the proliferation, invasion, and tumorigenicity of CSCs. Furthermore, activated FXR prominently upregulated the expression of SOCS3 while suppressing STAT3 phosphorylation in CSCs. To further investigate this discovery, we established a DEN-induced HCC model in mice and observed that FXR-deficient mice exhibited heightened susceptibility to HCC. This was accompanied by decreased expression levels of SOCS3 and elevated expression and phosphorylation levels of STAT3, as well as significantly enhanced HCC CSCs markers and stemness-related genes expression in DEN-induced HCC tissues of FXR-deficient mice. Additionally, we also found a significant upregulation of CSCs markers and stemness-related genes within HCC clinical samples. Based on these findings, we postulated that targeted regulation of SOCS3 by FXR inhibits STAT3 phosphorylation, thereby exerting an inhibitory effect on CSCs.

## 1. Introduction

Primary liver cancer is the sixth most commonly diagnosed cancer and the third leading cause of cancer death globally [1]. Although the precise mechanism of liver cancer remains unclear, evidence from leukemia, germ cell tumors, and various solid tumors supports the concept that cancer is sustained by a small population of self-renewing cancer stem cells (CSCs), commonly referred to as the CSCs model [2,3,4]. CSCs play a crucial role in tumor initiation, progression, invasion, metastasis, recurrence, and drug resistance [5]. Numerous studies have focused on liver cancer stem cells and proposed several molecules as markers for these cells, including CD133 [6,7], CD44 [8], CD90 [9], EpCAM [10], and CD24, etc. [11].

Chronic liver injury caused by cirrhosis or hepatitis is a crucial factor in the development of liver cancer [12]. Dysfunction in bile acid metabolism plays a significant role in chronic liver injury. Farnesoid X receptor (FXR, NR1H4), identified as an orphan nuclear receptor activated by farnesol, has been recognized since 1995. It serves as an intermediary in the mevalonate metabolic pathway [13]. FXR exhibits high expression levels in the liver, intestine, kidney, and adrenal glands and regulates bile acid synthesis, secretion, and reabsorption through feedback mechanisms [14]. Several bile acids, such as chenodeoxycholic acid (CDCA), cholic acid (CA), and deoxycholic acid (DCA), can activate FXR [15]. FXR is closely associated with the progression of HCC, and the mRNA level of FXR was found to be significantly decreased in human liver cancer tissues compared to normal liver tissues [16,17]. Some publications have also confirmed a significant decrease in FXR expression in HCC compared to normal tissues, which was found to be associated with HNF1a and β-catenin proteins [18,19]. Kim et al. demonstrated that FXR-deficient mice exhibited a predisposition to liver injury, characterized by an enhanced expression of pro-inflammatory factors, ultimately leading to an elevated susceptibility to liver cancer [20]. Similarly, Yang et al. observed a heightened occurrence of spontaneous liver cancer in FXR-deficient mice at 12 months of age [21]. The precise mechanism underlying the development of spontaneous tumors in FXR knockout mice remains elusive. Our previous findings indicated that FXR exerts hepatoprotective effects by suppressing hepatitis through the inhibition of the NF-κB signaling pathway, thereby impeding the onset of liver cancer [22]. Furthermore, numerous studies have revealed that, aside from inhibiting hepatic carcinogenesis, FXR also exhibits antagonistic effects against colon cancer, intestinal tumors, bile duct cancer, and gastroesophageal cancer [23,24,25,26].

Signal transduction and transcription activator 3 (STAT3), the most extensively studied member of the STAT family, exhibits hyperactivation in a majority of human cancers and is generally associated with poor clinical prognosis [27], highlighting its significance in cancer. Furthermore, increasing evidence has demonstrated that STAT3 is closely associated with CSCs [28]. The direct downstream genes of STAT3, including FOXM1, NANOG, TWIST, SLUG, and ZEB1, are linked to cancer stemness and EMT [29,30]. Additionally, studies have found that FXR inhibits the phosphorylation of STAT3 by upregulating the expression of SOCS3, thereby suppressing digestive system tumors [31,32,33].

However, the specific function and mechanisms of FXR in HCC stem cells remain unexplored. Our findings indicated that FXR was downregulated in HCC stem cells, and activating FXR significantly enhanced its inhibitory effects on CSCs, which may be related to the suppression of STAT3 phosphorylation. This discovery represents the first elucidation of the mechanism by which FXR regulated CSCs, highlighting a potential pathway to suppress the aggressive nature of HCC driven by CSCs. This finding paves the way for novel therapeutic strategies targeting FXR through the development of drugs that either mimic or enhance FXR activity, thereby modulating STAT3 activity. Such approaches could complement existing cancer treatments and improve outcomes for patients with liver cancer.

## 2. Results

### 2.1. The Expression of FXR Was Downregulated in HCC

**Figure 1:**
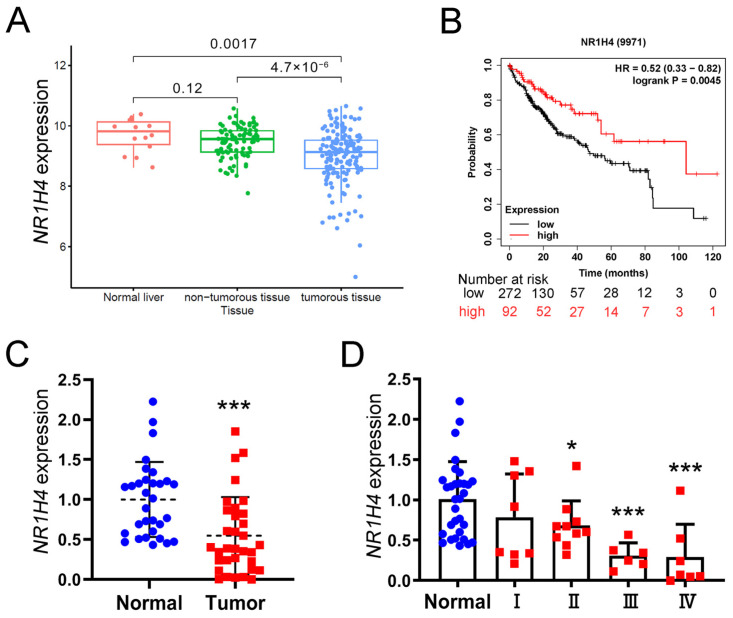
Expression and prognostic significance of FXR in HCC tissues. (**A**) FXR expression in HCC, adjacent tissues, and liver, according to the datasets of GSE102079. (**B**) Kaplan–Meier analysis was applied to demonstrate the correlation between FXR expression and overall survival of HCC patients. The cutoff for distinguishing low or high FXR expression was the median value. (**C**) FXR expression in HCC tissues and normal liver tissues were examined by qRT-PCR (*n* = 35). (**D**) Comparison of FXR mRNA levels in HCC patients, according to normal and I–IV stages (*n* = 35). All quantitative data are shown as means *±* SD, * *p* < 0.05, *** *p* < 0.001. vs. normal group, unpaired *t*-test.

Through analysis of the TIMER2 database, we observed a significant downregulation of FXR (*NR1H41*) in HCC compared to normal liver tissues, suggesting a potential negative correlation between *NR1H41* and the malignancy of HCC (Figure 1A). Furthermore, patients with a high expression of *NR1H41* have prolonged OS compared to those with a low expression of *NR1H41* (Figure 1B). To validate these findings, we performed qRT-PCR analysis of a cohort of 40 paired clinical HCC samples, which yielded consistent results with our previous analyses (Figure 1C,D).

### 2.2. Identification of CSCs in HCC Cell Lines

**Figure 2:**
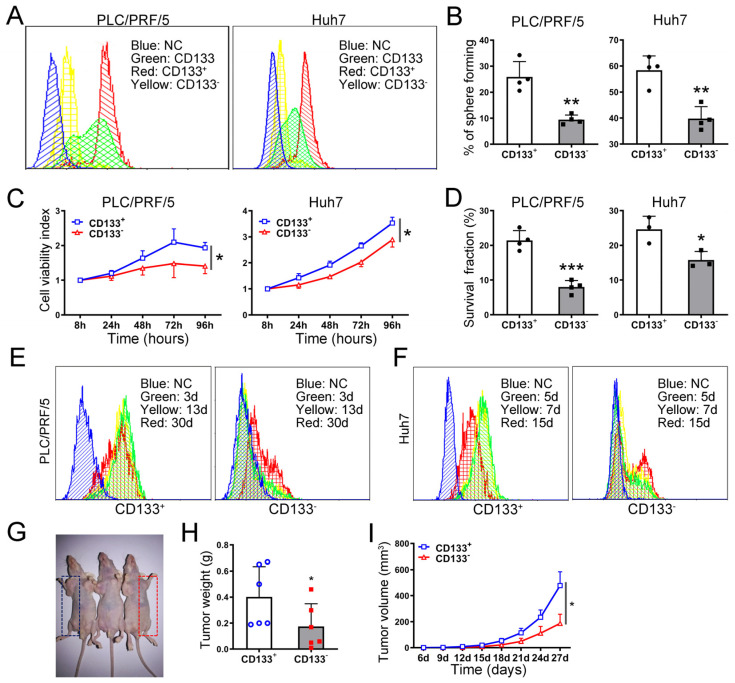
Identification of CSCs in HCC cell lines. (**A**) Efficiency of flow sorting of CD133^+^ and CD133^−^ cells in HCC cell lines. The effect of CD133^+^ and CD133^−^ cells on sphere formation (**B**), cell proliferation (**C**), clone formation (**D**), and cell differentiation (**E**,**F**) of HCC cell lines in vitro. The effect of CD133^+^ and CD133^−^ cells on HCC tumor growth in nude mice, representative tumor-bearing nude mice (**G**), tumor weight after sacrifice (**H**), and real-time tumor size (**I**) are shown. All quantitative data are represented as means ± SD, Smaller * *p* ≤ 0.1, * *p* ≤ 0.05, ** *p* ≤ 0.01, *** *p* ≤ 0.001. vs. CD133^+^ cells group, unpaired *t*-test.

The HCC cell lines used in this study, including HepG2, Huh7, Hep3B, and PLC/PRF/5, were employed as experimental models. The labeling of tumor cells can be achieved by utilizing HCC stem cell markers, including CD133, CD326, CD90, CD44, and CD338, as reported in the previous literature. Considering the significant differential expression of these markers among different HCC cell lines, our evaluation revealed that CD133 was widely expressed in various HCC cell lines, with notably higher expression levels in serum-free tumor spheres (Supplementary Figure S1). Therefore, we selected CD133 as a potential marker for further verification of HCC stem cells. Flow cytometry was utilized to sort out both the CD133^+^ and CD133^−^ populations from HepG2, Huh7, and PLC/PRF/5 HCC cell lines, respectively, while ensuring a purity level above 95% (Figure 2A; Supplementary Figure S2A).

Colony formation assay and CCK8 assay demonstrated that the survival and proliferation abilities of CD133^+^ cells were significantly stronger compared to that of CD133^−^ cells (Figure 2C,D; Supplementary Figure S2C,E). Moreover, spheroid formation assay revealed that the tumorigenic ability of CD133^+^ cells was markedly higher than that of the corresponding CD133^−^ population. The spheroids formed by CD133^+^ cells exhibited a higher formation rate along with larger volume size characterized by tighter cellular connections and clearer boundaries when compared to those formed by their negative counterparts (Figure 2B; Supplementary Figure S2B,F). Additionally, the CD133^+^ and CD133^−^ cells were continuously cultured, and the expression of CD133 was regularly assessed using flow cytometry. Differentiation potential was observed in both CD133^+^ and CD133^−^ cells, however, CD133^+^ cells exhibited a higher differentiation ability and a more rapid return to their initial state (Figure 2E,F, Supplementary Figure S2D). Subsequently, the xenotransplantation of CD133^+^ and CD133^−^ cells isolated from the PLC/PRF/5 cell line into nude mice revealed that CD133^+^ cells possessed stronger tumorigenic capacity (Figure 2G–I). Therefore, we proposed the existence of CSCs in HCC lines with a suggestion to employ CD133 as a marker.

### 2.3. FXR Was Lowly Expressed in HCC Stem Cells

**Figure 3:**
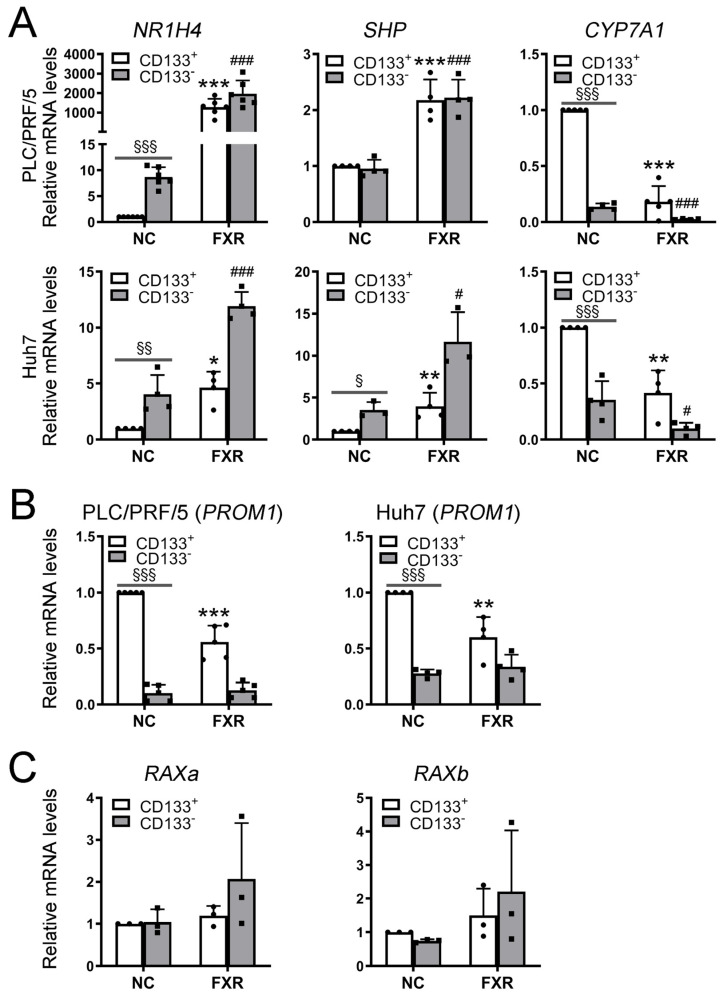
FXR is lowly expressed in HCC stem cells. (**A**) *NR1H4* and its target genes *SHP* and *CYP7A1* expression in CD133^+^ and CD133^−^ cells after activation of FXR were examined by qRT-PCR. (**B**) *PROM1* expression in CD133^+^ and CD133^−^ cells after activation of FXR were examined by qRT-PCR. (**C**) *NR1H4* dimers *RAXa* and *RAXb* expression in CD133^+^ and CD133^−^ cells after activation of FXR were examined by qRT-PCR. Data are presented as representative results of three independent experiments and are shown as the means ± SD. * *p* ≤ 0.05, ** *p* ≤ 0.01, *** *p* ≤ 0.001, FXR+CD133^+^ cells group vs. CD133^+^ cells group; # *p* ≤ 0.05, ### *p* ≤ 0.001, FXR+CD133^−^ cells group vs. CD133^−^ cells group; § *p* ≤ 0.05, §§ *p* ≤ 0.01, §§§ *p* ≤ 0.001, CD133^+^ cells group vs. CD133^−^ cells group, unpaired *t*-test.

The expression of FXR was found to be significantly downregulated in CD133^+^ cells sorted from Huh7 and PLC/PRF/5 HCC lines using flow cytometry. The downregulation of FXR was accompanied by the downregulation of its target gene *SHP* and the upregulation of its negative regulatory target gene *CYP7A1* (Figure 3A). Interestingly, the overexpression of FXR led to a decrease in CD133 gene (*PROM1*) expression, specifically in CD133^+^ cells, while having little effect on CD133^−^ cells (Figure 3B). Moreover, no differential expressions were observed for the dimers of FXR, including *RAXa* and *RAXb*, between CD133^+^ and CD133^−^ cells. Furthermore, the overexpression of FXR did not significantly affect the expression levels of *RAXa* and *RAXb* (Figure 3C).

### 2.4. FXR Suppressed the Malignant Phenotypes of CSCs In Vitro and In Vivo

**Figure 4:**
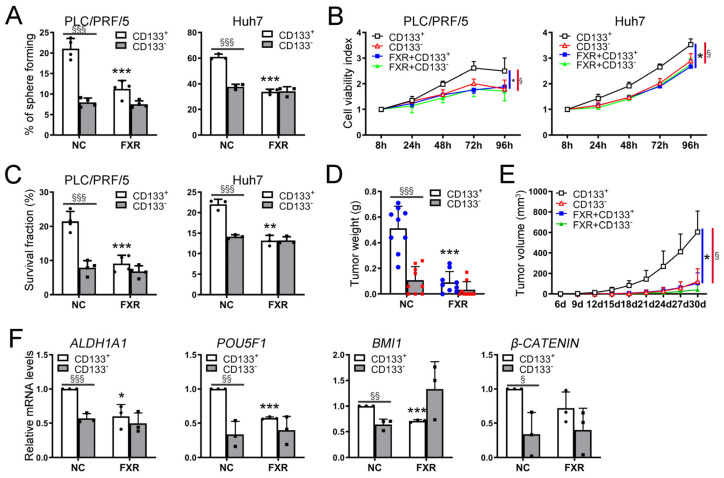
FXR suppresses the malignant phenotypes of CSCs in vitro and in vivo. The effect of CD133^+^ and CD133^−^ cells after activation of FXR on sphere formation (**A**), cell proliferation (**B**), and clone formation (**C**) of HCC cell lines in vitro. Xenograft tumors formed by CD133^+^ and CD133^−^ cells of PLC/PRF/5 cells after activation of FXR, tumor weight after sacrifice (**D**), and real-time tumor size (**E**) are shown. (**F**) Stemness-related gene expression in CD133^+^ and CD133^−^ cells of PLC/PRF/5 cells after activation of FXR were examined by qRT-PCR. All quantitative data are represented as means ± SD, Smaller * *p* ≤ 0.1, * *p* ≤ 0.05, ** *p* ≤ 0.01, *** *p* ≤ 0.001, FXR+CD133^+^ cells group vs. CD133^+^ cells group; Smaller § *p* ≤ 0.1, § *p* ≤ 0.05, §§ *p* ≤ 0.01, §§§ *p* ≤ 0.001, CD133^+^ cells group vs. CD133^−^ cells group; unpaired *t*-test.

Based on these findings, we postulated that FXR might play an important role in the functionality of CSCs. HepG2 cells were subjected to various concentrations of the FXR agonist CDCA, overexpression of FXR alone, and the combined overexpression of FXR with CDCA. The spheroid formation assay was employed to assess the effect of activating FXR on CSCs. Notably, all forms of activated FXR treatments significantly impeded tumor sphere formation, particularly when combining the overexpression of FXR with CDCA (Supplementary Figure S3). Furthermore, considering the low expression level of FXR in CSCs, we proceeded by overexpressing FXR and combining the agonist CDCA (50 μM) for subsequent experiments. The activation status of FXR was confirmed through qRT-PCR analysis examining the expression levels of FXR and its target genes *SHP* and *CYP7A1*. The results demonstrated that upon activation of FXR, there was a significant increase in FXR expression in both CD133^+^ and CD133^−^ cells, accompanied by the upregulation of *SHP* expression and the downregulation of *CYP7A1* expression (Figure 3A). To further investigate the impact of FXR activation on CSCs, we sorted CD133^+^ and CD133^−^ cells from the HepG2, Huh7, and PLC/PRF/5 HCC cell lines using flow cytometry. FXR was activated by overexpressing FXR combined with CDCA treatment. The spheroid formation assay revealed a significant reduction in tumor-sphere-forming capacity in CD133^+^ cells following FXR activation, whereas no significant changes were observed in CD133^−^ cells (Figure 4A, Supplementary Figure S4A). Furthermore, colony formation and CCK8 assays revealed a substantial decrease in survival and proliferation of CD133^+^ cells after FXR activation, whereas no significant inhibitory effect was observed on CD133^−^ cells (Figure 4B,C). Animal xenograft experiments also showed that activated FXR significantly inhibited the tumorigenic ability of CD133^+^ cells (Figure 4D,E; Supplementary Figure S4B). Moreover, high expression levels of stemness-related genes, including *ALDH1A1*, *POU5F1*, *BMI1*, and *β-CATENIN*, were detected in CD133^+^ cells through qRT-PCR analysis. The activation of FXR resulted in a notable downregulation of these stemness-related genes, specifically in CD133^+^ cells, while showing no significant effect on CD133^−^ cells (Figure 4F, Supplementary Figure S4C). These findings indicated that activated FXR exerted a more pronounced inhibitory effect on CSCs.

### 2.5. FXR Inhibited CSCs by Inhibiting STAT3 Phosphorylation Through SOCS3

**Figure 5:**
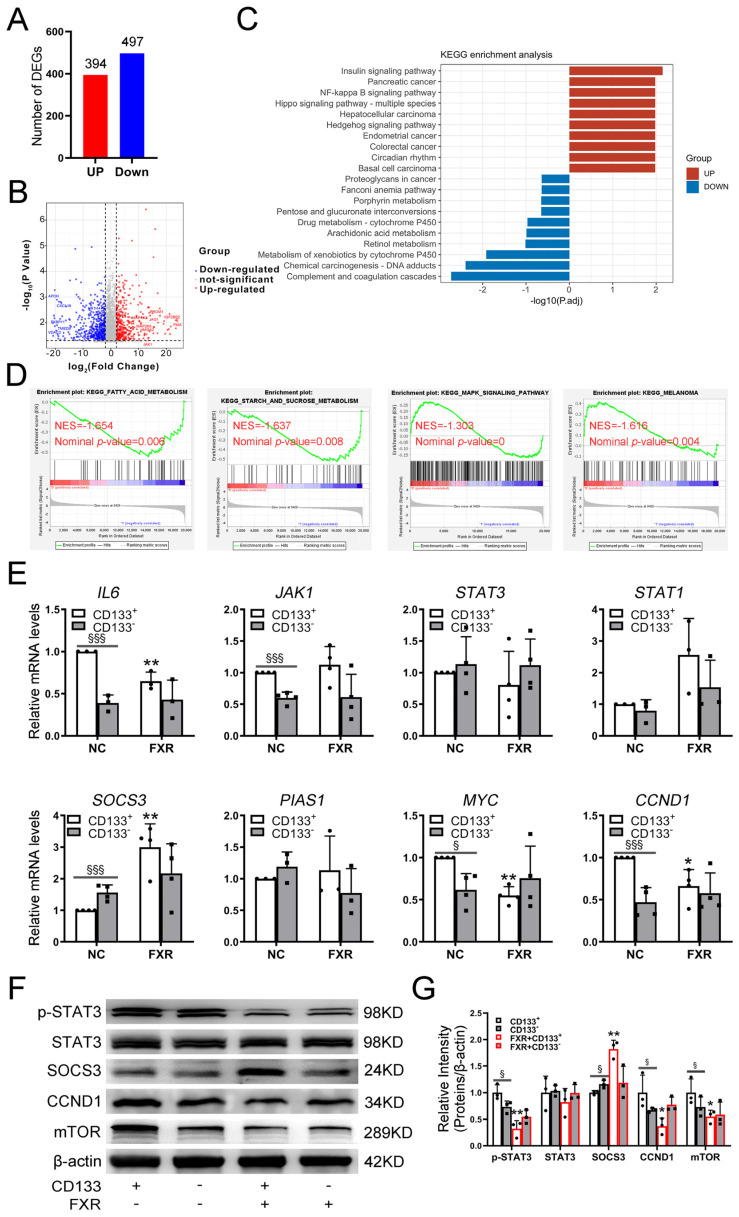
FXR inhibits CSCs by inhibiting STAT3 phosphorylation through SOCS3. (**A**) The numbers of differentially expressed genes between CD133^+^ and CD133^−^ cells in PLC/PRF/5 cells. (**B**) A volcano plot was generated to visualize the differential expression of genes, exhibiting a fold change equal to or greater than twofold between CD133^+^ and CD133^−^ cells in PLC/PRF/5 cells. (**C**) Bi-directional KEGG functional enrichment analysis of the differentially expressed genes using the Hiplot database. Terms with up/downregulated genes are shown in red/blue, respectively. (**D**) GSEA enrichment plot of KEGG pathway for the RNA-seq dataset of CD133^+^ and CD133^−^ cells. The FDR estimates the statistical significance of a pathway enrichment score. (**E**) IL6/JAK/STAT signaling pathway related gene expression in CD133^+^ and CD133^−^ cells of PLC/PRF/5 cells after activation of FXR were examined by qRT- PCR. (**F**,**G**) Western blotting and densitometric quantitative analysis of STAT3, p-STAT3, SOCS3, CCND1, and mTOR in response to CD133^+^ and CD133^−^ cells of PLC/PRF/5 cells after activation of FXR, where β-actin served as the internal control. Data are presented as representative results of three independent experiments and are shown as the means ± SD. * *p* ≤ 0.05, ** *p* ≤ 0.01, FXR+CD133^+^ cells group vs. CD133^+^ cells group; § *p* ≤ 0.05, §§§ *p* ≤ 0.001, CD133^+^ cells group vs. CD133^−^ cells group, unpaired *t*-test.

To further investigate the mechanism by which FXR inhibits CSCs, RNA sequencing was conducted on CD133^+^ and CD133^−^ cells. The results revealed that, in comparison to CD133^−^ cells, a total of 891 differentially expressed genes were identified in CD133^+^ cells, with 394 genes being upregulated and 497 genes being downregulated (|logFC| ≥ 2, *p* ≤ 0.05) (Figure 5A). The expression level of *PROM1* was notably upregulated in CD133^+^ cells, whereas *NR1H4* exhibited significant downregulation, which confirmed the reliability of the transcriptome sequencing data (Figure 5B). Additionally, we conducted bi-directional KEGG functional enrichment analysis of these differentially expressed genes using the Hiplot database. Furthermore, GSEA analysis was conducted based on our RNA-seq dataset. These findings demonstrated that the upregulated genes in CD133^+^ cells primarily participated in tumorigenesis, proliferation, and inflammation pathways, whereas the downregulated genes were involved in lipid metabolism, as well as amino-acid-metabolism- and glucose-metabolism-related pathways (Figure 5C,D). These results further validated the enhanced proliferative capacity and malignant potential of CD133^+^ cells as CSCs. Additionally, the results highlighted the elevated expression of FXR in CD133^−^ cells, which regulates metabolic pathways associated with lipid metabolism, amino acid metabolism, and glucose metabolism.

Interestingly, we further confirmed that the IL6/JAK/STAT signaling pathway was overactivated in the CD133^+^ cell subset. Compared to CD133^−^ cells, CD133^+^ cells exhibited significant upregulation in the expression levels of *IL6*, *JAK1*, *MYC*, and *CCND1*. In contrast, no significant changes were observed in the expression levels of *STAT3*, *STAT1*, and *PIAS1*. Upon activation of FXR in CD133^+^ cells, significant downregulation was observed in the expression levels of *IL6*, *MYC*, and *CCND1*. In contrast, the expression levels of *JAK1*, *STAT3*, *STAT1*, and *PIAS1* remained unchanged. It is worth noting that *SOCS3*, as a target gene of FXR, exhibited high expression in CD133^−^ cells. However, after the activation of FXR, there was a more significant upregulation of *SOCS3* in CD133^+^ cells compared to CD133^−^ cells (Figure 5E). The western blot experiments showed consistent results, indicating that although FXR did not affect the overall protein expression of STAT3, it specifically enhanced the phosphorylation level of STAT3 within the CD133^+^ cell subset. Upon activation of FXR, the phosphorylation of STAT3 was specifically inhibited in the CD133^+^ cell subset, but not in CD133^−^ cells. This finding was further corroborated by the altered protein expression levels of CCND1 and mTOR, both of which were established downstream targets of activated STAT3. These genes exhibited upregulation in CD133^+^ cells compared to CD133^−^ cells yet exhibited downregulation following FXR activation. Furthermore, consistent with the qRT-PCR results, the western blot experiments confirmed that the activation of FXR resulted in a more pronounced upregulation of SOCS3 in CD133^+^ cells (Figure 5F,G). Based on previous studies indicating that SOCS3 can inhibit IL6/JAK/STAT signaling by suppressing phosphorylation of STAT3 [31,32], we hypothesized that the activation of FXR inhibits CSCs by suppressing STAT3 phosphorylation via SOCS3.

### 2.6. The Deficiency of FXR Led to the Downregulation of SOCS3 Expression, Resulting in Increased STAT3 Phosphorylation and Promotion of Primary Liver Cancer Development

**Figure 6:**
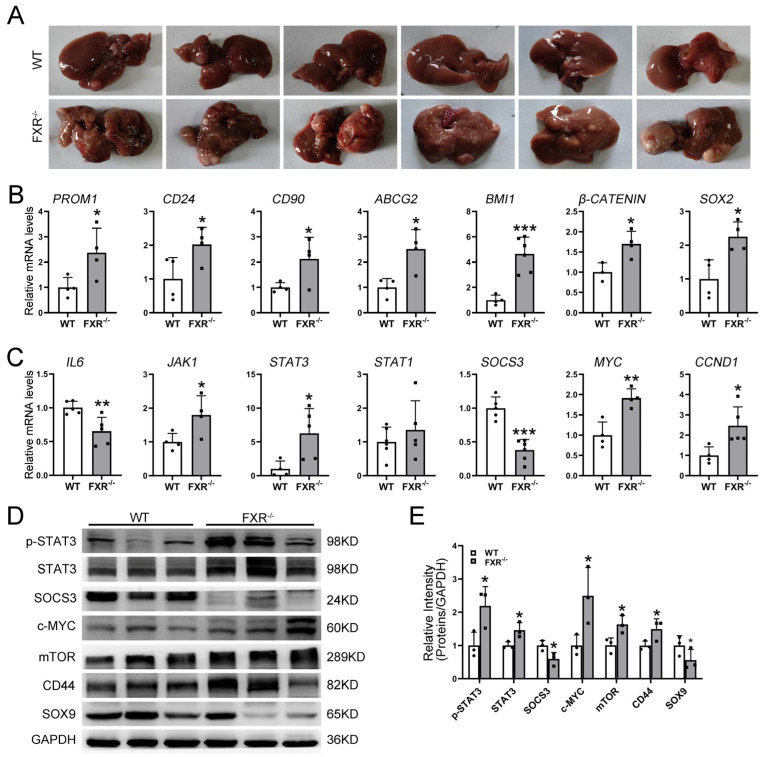
The deficiency of FXR leads to the downregulation of SOCS3 expression, resulting in reduced inhibition of STAT3 phosphorylation and promotion of primary liver cancer development in the DEN-induced mouse model. (**A**) Representative images of liver from WT and FXR^−/−^ mice in DEN models. (**B**) CSCs markers and stemness-related gene expression in the liver from WT and FXR^−/−^ mice in DEN models were examined by qRT-PCR. (**C**) IL6/JAK/STAT signaling-pathway-related gene expression in the livers of WT and FXR^−/−^ mice in DEN models were examined by qRT-PCR. (**D**,**E**) Western blotting and densitometric quantitative analysis of STAT3, p-STAT3, SOCS3, c-MYC, mTOR, CD44, and SOX9 in liver from WT and FXR^−/−^ mice in DEN models. GAPDH served as the internal control. Data are shown as the means ± SD. Smaller * *p* ≤ 0.1, * *p* ≤ 0.05, ** *p* ≤ 0.01, *** *p* ≤ 0.001, WT group vs. FXR^−/−^ group, unpaired *t*-test.

In order to investigate the correlation between FXR and HCC occurrence, we established a DEN-induced HCC model. Consistent with previous reports [20,21], the incidence of liver cancer was significantly higher in FXR^−/−^ mice compared to WT mice (Figure 6A). To further explore the association between FXR and CSCs, we examined the expression of CSCs markers and stemness-related genes in the DEN HCC model. We observed a significant upregulation of CSCs markers such as *CD133*, *CD90*, and *CD24*, as well as stemness genes including *BMI1*, *SOX2*, and *β-CATENIN* in FXR^−/−^ mice (Figure 6B). Additionally, the activation of the IL6/JAK/STAT signaling pathway was more pronounced in the livers of FXR^−/−^ mice compared to those of WT mice. This was accompanied by a significant upregulation in the expression of STAT3 target genes, including *C-myc* and *CCNB1*, while simultaneously downregulating the levels of *SOCS3* and *IL6* (Figure 6C). Western blot analysis also confirmed heightened activation of the IL6/JAK/STAT signaling pathway in FXR^−/−^ mouse livers. Notably, both the expression level and phosphorylation of STAT3 were significantly elevated in the livers of FXR^−/−^ mice, coinciding with an upregulation of the STAT3 target genes C-myc and mTOR. Conversely, the expression of SOCS3 was suppressed. Furthermore, there was a notable elevation in CSCs marker CD44 but decreased levels of SOX9 in FXR^−/−^ mice (Figure 6D,E). Therefore, our findings suggested that reduced SOCS3 expression coupled with enhanced STAT3 phosphorylation activated the IL6/JAK/STAT signaling pathway, resulting in an elevated incidence of primary liver cancer in FXR^−/−^ mice. Given the critical role of STAT3 in maintaining CSCs stemness, the dysregulation of this pathway may lead to an elevated expression of CSCs markers and stemness-associated genes in FXR^−/−^ mouse livers.

### 2.7. SOCS3 and FXR Are Positively Correlated and Lowly Expressed in HCC

**Figure 7:**
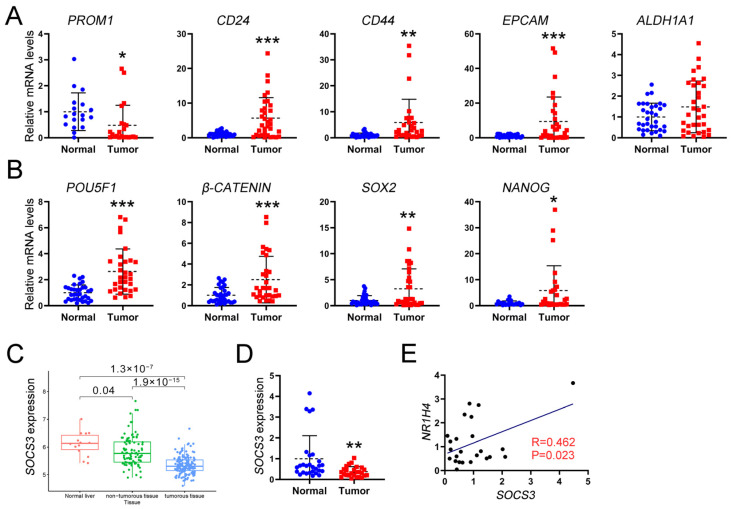
FXR inhibits CSCs stemness by targeting SOCS3. (**A**) CSCs markers and (**B**) stemness-related gene expression in HCC tissues and normal liver tissues was examined by qRT-PCR (*n* = 35). (**C**) *SOCS3* expression in HCC, adjacent tissues, and liver, according to the dataset GSE102079. (**D**) *SOCS3* expression in HCC tissues and normal liver tissues was examined by qRT- PCR (*n* = 35). (**E**) The correlation between *SOCS3* and *NR1H4* in HCC was determined by Pearson analysis. All data are shown as the means ± SD. * *p* ≤ 0.05, ** *p* ≤ 0.01, *** *p* ≤ 0.001. vs. normal group, unpaired *t*-test.

The qRT-PCR analysis of clinical HCC samples revealed a significant upregulation of CSCs markers, including *CD24*, *EPCAM*, *CD44*, and *ALDH1A1*, as well as stemness-associated genes such as *POU5F1*, *SOX2*, and *NANOG* in HCC tissues compared to normal liver tissues (Figure 7A,B). Pearson correlation analysis was employed to examine the relationship between FXR and these CSCs markers, as well as stemness genes. The results revealed a weak negative correlation between FXR and the expression levels of *CD44* or *CD24*, while showing a positive correlation between FXR and NANOG. No significant correlation was observed between FXR and *PROM1*, *EPCAM*, *POU5F1*, *ALDH1A1*, *SOX2*, or *β-CATENIN* (Supplementary Figure S5A).

Based on the TIMER database, we observed a significant downregulation of *SOCS3* in liver cancer tissues compared to normal liver tissues. Moreover, we found a negative correlation between *SOCS3* expression and the malignancy grade of liver cancer, whereas a positive correlation was observed between *SOCS3* expression and FXR levels (Figure 7C, Supplementary Figure S5B). Additionally, qRT-PCR analysis of clinical HCC samples confirmed our previous findings showing that the expression of *SOCS3* was significantly downregulated in HCC and exhibited a positive correlation with FXR (Figure 7D,E). Furthermore, no statistically significant associations were identified between SOCS3 and CSCs markers or stemness-related genes (Supplementary Figure S5C). These results suggested that FXR regulated CSCs through its modulation of SOCS3.

## 3. Discussion

CSCs play a pivotal role in the initiation, progression, invasion, metastasis, recurrence, and acquisition of drug resistance in tumors [34,35]. In the study of CSCs, their isolation is an essential prerequisite. We performed an extensive evaluation of the expression profiles of established markers for HCC stem cells, including CD133, CD24, CD326, CD90, CD44, and CD338, in the HepG2, Huh7, and PLC/PRF/5 cell lines. Among these markers, CD133 was selected as the most reliable indicator for identifying HCC stem cells. CD133 is a membrane-bound pentaspan glycoprotein initially identified in murine neuroepithelial stem cells and subsequently detected in human tissues [36]. It has been widely used as a CSCs marker across various tumor types [37]. Some studies suggest that combining CD133 with other CSCs markers, such as CD44, provides an optimal CSCs-specific marker for liver and colorectal cancers [38,39]. We selected CD133 as a single marker, due to its consistent expression in HCC cell lines. Subsequent experiments confirmed that CD133 can serve as a robust marker for characterizing the HCC cell lines HepG2, Huh7, and PLC/PRF/5, which is consistent with previous findings [6,7,40,41,42].

FXR is recognized as a negative regulator of tumor progression [43] and has been shown to exhibit reduced expression in HCC [16,17,18,19]. Through bioinformatics analysis, we observed significantly lower FXR expression levels in HCC tissues compared to normal liver tissues. Moreover, this downregulation was negatively correlated with the malignancy grade of HCC. To substantiate these observations, we conducted experiments using clinical samples from HCC patients, evaluating FXR expression in both tumor and adjacent non-tumor tissues. The results confirmed our previous conclusion and further emphasized the role of FXR as a negative regulatory factor in HCC. Notably, when comparing gene expression profiles between CSCs (CD133^+^ cells) and non-CSCs (CD133^−^ cells) in the Huh7 and PLC/PRF/5 cell lines, we observed a significantly lower expression of FXR in CSCs. This represents the first report of differential FXR expression in HCC CSCs, despite previous studies indicating that the selective activation of intestinal FXR can inhibit the abnormal proliferation of Lgr5^+^ cells and impede colorectal cancer progression. Additionally, FXR regulates asymmetric cell division of Sox9^+^ cells via the Notch1 pathway to prevent liver cancer development [44,45]. This raises the question of whether the tumor suppressor effect of FXR is associated with CSCs. To further investigate this phenomenon, we independently activated FXR in both CSCs and non-CSCs tumor cells. The results demonstrated that FXR activation markedly reduced the survival rates, proliferation rates, and tumorigenic abilities, specifically in CD133^+^ cells. In contrast, no significant inhibitory effects were observed in CD133^−^ cells. The differential expression levels of FXR in CD133^+^ cells compared to CD133^−^ cells may contribute to this observed disparity. Specifically, the higher expression of FXR in CD133^−^ cells may mitigate the inhibitory effects of FXR activation. These findings provide additional evidence supporting our hypothesis that FXR plays a more pronounced role in suppressing CSCs.

An increasing number of studies have demonstrated the crucial role of STAT3 in maintaining the stemness of CSCs [28,29,30]. Our findings indicated that the IL6/JAK/STAT signaling pathway is excessively activated in the CD133^+^ cell subset. Notably, upon FXR activation, STAT3 phosphorylation is significantly inhibited, specifically in CD133^+^ cells, but not in CD133^−^ cells. This suggested a potential mechanism by which FXR may exert its anticancer effects through the selective suppression of STAT3 phosphorylation in CSCs. Furthermore, we identified SOCS3 as a downstream target gene regulated by FXR. Importantly, our data demonstrate a downregulation of SOCS3 in CD133^+^ cells, which was significantly restored upon FXR activation. Previous reports have demonstrated that FXR inhibits STAT3 phosphorylation by upregulating SOCS3 expression, thereby suppressing digestive system tumors [31,32,33]. Herein, we further confirmed that, in HCC stem cells, FXR activation leads to increased SOCS3 expression and the subsequent inhibition of STAT3 phosphorylation. Current studies have elucidated two distinct mechanisms through which SOCS3 modulates the JAK signaling pathway. The first mechanism involves the direct inhibition of JAK kinase activity, thereby preventing the activation of STAT proteins. SOCS3 is a JAK-binding protein that has been demonstrated to inhibit JAK2 kinase activity by directly binding to the activation loop through the SH2 domain [46,47]. The second mechanism involves the indirect modulation of JAK proteins. IL6 activates both the Jak/STAT pathway and the mitogen-activated protein kinase cascade. Tyrosine 759 of the IL6 signal-transducing receptor subunit gp130 has been identified as a key site for the negative regulation of IL6-induced gene expression and activation of the Jak/STAT pathway. Additionally, it has been reported that SOCS3 contributes to the Tyr-759-dependent attenuation of IL6 signaling through gp130 [48]. SOCS3 contains a region known as the SOCS box, which can recruit E3 ubiquitin ligases. In 2014, Kershaw et al. reported that SOCS3 catalyzes the ubiquitination of both gp130 and JAK2 [49]. Therefore, we concluded that FXR suppresses STAT3 phosphorylation via SOCS3-mediated mechanisms in HCC stem cells. It is noteworthy that the latest report has revealed that FXR promotes non-small-cell lung cancer metastasis by activating the Jak2/STAT3 signaling pathway through the transactivation of the IL6ST and IL6 genes [50]. These findings appear to contradict the tumor-suppressive role of FXR observed in digestive system tumors. This discrepancy may be attributed to the tissue-specific effects of FXR. Previous studies have confirmed that FXR promotes the carcinogenesis of non-small-cell lung cancer [51,52], highlighting the intricate role of FXR in cancer biology.

FXR deficiency in mice results in the increased proliferation of colon cells and spontaneous liver tumors [21,53]. DEN exposure triggers inflammatory responses in liver tissues, which subsequently leads to the development of HCC. Through the study of DEN-induced animal models, researchers have gained valuable insights into the mechanisms underlying hepatocarcinogenesis. Previous studies using these models have highlighted the critical role of Lgr5^+^ stem cells in hepatocarcinogenesis [54]. The PTEN/AKT and Wnt/β-catenin pathways play key roles in regulating the proliferation of Lgr5^+^ cells, offering important clues for potential therapeutic strategies. Furthermore, Lgr5 has emerged as a promising target for antibody-based or drug-delivery treatments [55]. Therefore, we also explored the regulatory effect of FXR on CSCs based on the DEN-induced animal models of HCC. In the DEN HCC model, we observed a significantly higher incidence of HCC in FXR^−/−^ mice compared to WT mice. This was accompanied by elevated levels of STAT3 phosphorylation in the livers of FXR^−/−^ mice. Consequently, our findings supported the conclusion that FXR inhibits HCC development by suppressing STAT3 phosphorylation via SOCS3-mediated inhibition. Furthermore, to elucidate the correlation between FXR and CSCs, we assessed the expression levels of CSCs markers and stemness-related genes in the DEN-induced HCC model. Our analysis revealed a significant upregulation of these genes in FXR^−/−^ mice. Considering the critical role of STAT3 in sustaining CSCs stemness properties, a lack of FXR activation leads to sustained activation of STAT3 and maintenance of CSCs stemness characteristics. Therefore, our study proposed that FXR functions as a critical suppressor of CSCs. We also performed qRT-PCR and bioinformatics analyses on clinical HCC samples. Our results demonstrated a significant downregulation of SOCS3 expression in HCC tissues, which was positively correlated with the expression of FXR. These results further emphasized that FXR suppressed the malignant characteristics of CSCs by inhibiting STAT3 phosphorylation via the targeted modulation of SOCS3. The mechanism provides valuable insights into the complex interplay between FXR and intracellular signaling pathways during cancer development, potentially paving the way for novel therapeutic strategies targeting CSCs. Additionally, future studies investigating how FXR modulates SOCS3 and consequently impacts STAT3 signaling in CSCs could facilitate the development of more effective treatments aimed at disrupting the CSCs, ultimately improving patient outcomes across various cancer types. Furthermore, it would be valuable to examine the long-term outcomes of FXR activation across different stages of HCC therapy, as well as any potential side effects or resistance mechanisms that might emerge. Moreover, preclinical models such as patient-derived xenografts, tumor organoids, and 3D bioprinting can offer crucial information about the efficacy and safety of FXR activators. Additionally, clinical trials designed to evaluate the synergistic effects of FXR activation in combination with existing treatments like chemotherapy or targeted therapies would enhance our understanding and guide the advancement of HCC therapy.

**Figure 8:**
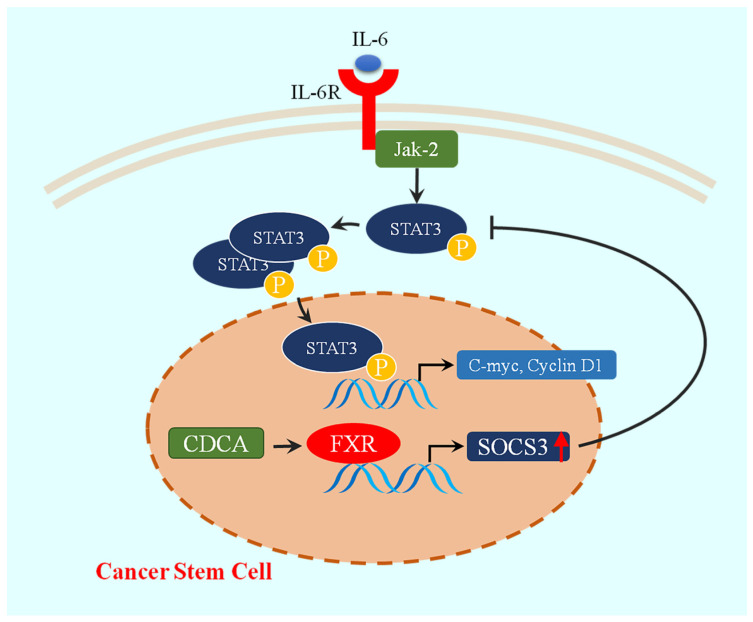
A proposed model for the tumor-suppressive activity of FXR. The red arrow indicates the upregulation of SOCS3.

In conclusion, our findings suggested that FXR exerted a tumor-suppressive effect in HCC by inhibiting the phosphorylation of STAT3 in HCC stem cells through targeting SOCS3 (Figure 8). This discovery represented the first elucidation of the underlying mechanism by which FXR regulated CSCs, thereby expanding our understanding of its role in tumor inhibition and providing a theoretical basis for clinical treatment of HCC, as well as the development of drugs targeting CSCs.

## 4. Materials and Methods

### 4.1. Reagents and Plasmids

The CDCA and DEN were acquired from Sigma Chemical (St. Louis, MO, USA). The FXR overexpression plasmid was obtained from GenePharma (Shanghai, China).

### 4.2. Collection of HCC Tissue Specimens

The human hepatocellular carcinoma (HCC) samples were obtained from the Department of General Surgery, Affiliated Huaihe Hospital of Henan University, from patients diagnosed with HCC. None of the patients received preoperative anti-cancer treatments. All tissues were snap-frozen immediately and always kept in liquid nitrogen until further analysis. The collection of HCC tissue samples and related clinical data was approved by the Biomedical Research Ethics Committee of Henan University, Kaifeng.

### 4.3. Cell Culture and Transfection

HCC cell lines HepG2, PLC/PRF/5, Huh7, Hep3B, and Hepa1-6 were obtained from the Institute of Basic Medical Sciences (IBMS) of the Chinese Academy of Medical Sciences. The cells were cultured in complete medium consisting of MEM/DMEM supplemented with 10% (*v*/*v*) heat-inactivated fetal calf serum and 1% (*v*/*v*) antibiotics-antimycotics. A total of 6 × 10^5^ cells were seeded in 60 mm culture dishes with complete medium. Transient transfection of cells with FXR expression plasmids or the empty plasmid (as a control) was performed using Lipofectamine 3000 (Invitrogen, Carlsbad, CA, USA). Twenty-four hours after transfection, the cells were pre-treated with CDCA at a concentration of 50 μM for one day. Subsequently, the cells were harvested for qRT-PCR or western blotting analysis.

### 4.4. The Model of Primary Hepatocellular Carcinoma

The wild-type (WT) and FXR-deficient (FXR^−/−^) male mice, on a C57BL/6J background from Merck Research Laboratories in Kenilworth, NJ, were housed in a pathogen-free animal facility following a standard 12 h light–dark cycle.

The 2-week-old WT mice and FXR^−/−^ mice were intraperitoneally injected with DEN (25 mg/kg) and followed a normal diet. At the 40th week, the mice were anesthetized, and their liver tissues were either fixed or frozen at −80 °C for subsequent experiments. The expression of genes or proteins involved in the IL6/JAK/STAT signaling pathway, CSCs markers, and stemness genes was assessed using qRT-PCR or western blotting.

The animal experiments were conducted in compliance with the National Institute of Health Guide for the Care and Use of Laboratory Animals, following approval from the Biomedical Research Ethics Committee of Henan University, Kaifeng (HUSOM-2018-328).

### 4.5. RNA Extraction and Quantitative Real-Time PCR (qRT-PCR)

The extraction of total RNA from cells and tissues was performed using Tri-Reagent (Molecular Research Center, Inc., Cincinnati, OH, USA). Subsequently, qRT-PCR analysis was conducted following the Power SYBR Green PCR Master Mix protocol (Applied Biosystems, Foster City, CA, USA). The amplification of β-actin or 36B4 served as an internal reference. Quantitative PCR analysis was carried out utilizing the ABI 7500 Sequence Detection System.

### 4.6. Flow Cytometric Analysis and Fluorescence-Activated Cell Sorting (FACS)

The HCC cell lines and tumor spheres were prepared and labeled with conjugated anti-human CD133, CD44, CD326, CD90, and CD338 antibodies (Miltenyi Biotec, Gladbach, Germany). Isotype-matched immunoglobulin was used as a control. The cells were incubated for 15 min at 4 °C in the dark and then washed with PBS. Subsequently, the cells were resuspended in PBS and filtered through sterile 200-mesh cell strainers to obtain single cells. Flow cytometric analysis of the cells was performed using MoFlo XDP, according to the manufacturer’s protocol (Beckman, Indianapolis, IN, USA).

For the cell sorting experiments, HCC cell lines HepG2, PLC/PRF/5, and Huh7 were subjected to immunostaining with CD133 antibody (Miltenyi Biotec, Gladbach, Germany). Subsequently, the populations of CD133^+^ and CD133^−^ cells were sorted using MoFlo XDP. The selected cells were then cultured for propagation purposes, and the expression levels of CD133 were determined through flow cytometric analysis.

### 4.7. The Formation of Tumor Spheres

The HCC cells were suspended in DMEM/F12 medium supplemented with 20 ng/mL of epidermal growth factor (EGF, Thermo, Waltham, MA, USA), 20 ng/mL of basic fibroblast growth factor (bFGF, Thermo, USA), 10 ng/mL heparin (Sigma, St. Louis, MO, USA), B27 (1:50; Invitrogen), 1% methylcellulose (Sigma, USA), and 1% antibiotics to create a stem-cell-permissive medium. All cultures were maintained at a temperature of 37 °C in an atmosphere consisting of 5% CO_2_/95% air. Tumor spheres that formed within a period of ten days were observed and recorded under the microscope.

### 4.8. Cell Colony Formation Assay

The same number of cells were cultured in MEM media supplemented with 10% FBS in a 60 mm dish until visible colony formation occurred. Each treatment for each cell type was performed in triplicate. Cell colonies were fixed and stained with a solution containing 0.5% Toludine Blue O and 4% PFA. Colonies containing more than 50 cells were counted as survivors.

### 4.9. Cell Viability Assays

The cell proliferation assay was performed using a Cell Counting Kit-8 (Beyotime, Shanghai, China). Briefly, cells treated differently were seeded at a density of 7 × 10^3^ cells per well in 96-well plates with 100 µL of medium per well. The cells were cultured for various time points (8, 24, 48, 72, and 96 h), followed by the addition of 10 µL of Cell Counting Kit-8 (CCK-8) reagent to each well. After incubation for an additional period of two hours at a temperature of 37 °C, the absorbance at a wavelength of 450 nm was measured using a microplate reader (Enpire, PE, Tulsa, OK, USA).

### 4.10. Western Blot Analysis

Protein extraction from cells or tissues and immunoblotting were performed following our previously established protocols [56]. Specific primary antibodies against SOCS3, STAT3, p-STAT3, CCND1, mTOR, c-MYC, CD44, SOX9, GAPDH, and β-actin were utilized. All primary antibodies were procured from Cell Signaling Technology (Danvers, MA, USA). The blots were visualized using SuperSignal West Pico Chemiluminescent Substrates (Thermo Fisher Scientific Inc., USA), and the signals were captured using an Automatic Multifunction Chemiluminescent Detection System (Tanon Science & Technology Co., Ltd., Shanghai, China). The band intensities were quantified using Image-J Software (1.53t).

### 4.11. Xenograft Tumor Assay

The male BALB/c nude mice, aged 4 weeks, were obtained from Beijing Vital River Laboratories (Beijing, China) and maintained under specific pathogen-free (SPF) conditions with standard rodent chow. Subsequently, the mice received a subcutaneous injection of 2 × 10^6^ cells resuspended in 150 μL of serum-free PBS into their thigh region. Tumor volume was estimated by measuring tumor size every three days using a Vernier caliper (length × width × width). After a period of 30 days, the mice were anesthetized and sacrificed to collect all tumors.

### 4.12. RNA-Seq and Data Analysis

The populations of CD133^+^ and CD133^−^ cells were sorted from PLC/PRF/5 cells with MoFlo XDP. Total RNA of CD133^+^ and CD133^−^ cells were isolated using TRIzol Reagent and quantified with NanoDrop. RNA-seq analysis was conducted on a BGIseq500 platform (BGIShenzhen, Shenzhen, China). Firstly, SOAPnuke (v1.5.6) was used for quality trimming to remove low-quality bases and the sequencing adapter. Subsequently, the sequencing data were aligned to the human genome (human. GRCh38/hg38) using HISAT2 (v2.0.4), followed by aligning the clean reads to the reference coding gene set using Bowtie2 (v2.5.2). Furthermore, differentially expressed genes (DEGs) were analyzed with DESeq2 with *p*-value ≤ 0.05, |logFC| ≥ 1. To obtain insight to the change in phenotype, Bi-directional functional enrichment analysis of KEGG signaling pathways for DEGs was conducted with the Hiplot database (https://hiplot.com.cn/home/index.html, accessed on 15 February 2024. The GSEA software (4.3.2) was employed to screen potential regulatory pathways, and differential significance was determined based on a threshold of |normalized enrichment score (NES)| ≥ 1 and Q value ≤ 0.05.

### 4.13. Data Acquisition and Analysis

Expression profiling data of the GSE102079 dataset (14 normal live tissues, 91 non-tumorous tissue, and 152 live tumorous tissue) was obtained from the Gene Expression Omnibus (GEO) database. The correlation analysis of *NR1H4* or *SOCS3* mRNA expression and clinicopathological parameters of HCC patients were conducted using R package cliCor. Kaplan–Meier curves of OS were generated using the Kaplan–Meier Plotter database for prognosis analysis comparing patients with low or high NR1H4 expression. The correlation of two genes in LIHC tumor was computed using the TIMER2.0 database.

### 4.14. Statistical Analysis

Statistical significance was analyzed using SPSS 25.0 software, and figures were drawn using GraphPad Prism 8.0. Two-sided Student’s *t* test was conducted to evaluate the statistical significance of the difference between the two groups. The correlation was evaluated by Pearson correlation analysis, and the error bar indicates the standard deviation of the mean value. A *p*-value of less than 0.05 was considered statistically significant. One, two, and three asterisks represent *p*-values of < 0.05, 0.01, and 0.001, respectively.

## Data Availability

The data presented in this study are available in the article.

## Supplementary Materials

The following supporting information can be downloaded at: https://www.mdpi.com/article/10.3390/ijms26031122/s1.
