# Supplementary material for: Farnesoid X Receptor Attenuates the Tumorigenicity of Liver Cancer Stem Cells by Inhibiting STAT3 Phosphorylation
Source: Int J Mol Sci. 2025 Jan 28;26(3):1122. doi: 10.3390/ijms26031122 (PMC11817294; doi:10.3390/ijms26031122)
Supplement: Supplementary file 1 [file ijms-26-01122-s001.zip › ijms-3395717-supplementary materials captions.pdf]

International Journal of
Molecular Sciences

MDPI

Supplementary Materials:

Figure S1. Identification of CSCs markers in HCC cell lines. (**A**) Representative images of sphere formation in different HCC cell lines in vitro (Magnif. 100×). (**B**) Representative of flow cytometry results of CSCs markers in different HCC cell lines, the red line is the control and the green line is the antibody labeling. (**C**) Representative of flow cytometry results of CSCs markers in sphere of different HCC cell lines, the red line is the control and the green line is the antibody labeling.

Figure S2. Identification of CSCs in HCC cell lines. (**A**) Efficiency of flow sorting of $CD133^+$ and $CD133^-$ cells in HepG2 cells. The effect of $CD133^+$ and $CD133^-$ cells on sphere formation (**B**), cell proliferation (**C**) and cell differentiation (**D**) of HepG2 cells in vitro. (**E**) Representative images of clone formation by $CD133^+$ and $CD133^-$ cells of HCC cell lines. (**F**) Representative images of sphere formation by $CD133^+$ and $CD133^-$ cells of HCC cell lines (Magnif. 100×). $^*P \leqslant 0.1$, $^{**}P \leqslant 0.01$. vs. $CD133^+$ cells group.

Figure S3. Activation of FXR inhibited tumor sphere formation. (**A**) Effect of different treatments on activated FXR on sphere formation of HepG2 cells in vitro. (**B**) Representative images of different treatments on activated FXR on sphere formation of HepG2 cells (Magnif. 100×). $^*P \leqslant 0.05$, $^{**}P \leqslant 0.01$, $^{***}P \leqslant 0.001$. vs. NC.

Figure S4. FXR suppresses the malignant phenotypes of CSCs. (**A**) The effect of $CD133^+$ and $CD133^-$ cells after activation of FXR on sphere formation of HepG2 cells in vitro. (**B**) Representative dissected tumours formed by $CD133^+$ and $CD133^-$ cells of PLC/PRF/5 cells after activation of FXR from nude mice were shown. (**C**) Stemness-related genes expression in $CD133^+$ and $CD133^-$ cells of Huh7 cells after activation of FXR were examined by qRT-PCR. $^*P \leqslant 0.1$, $^*P \leqslant 0.05$, $^{**}P \leqslant 0.01$, FXR+$CD133^+$ cells group vs. $CD133^+$ cells group. §$P \leqslant 0.05$, §§$P \leqslant 0.01$, $CD133^+$ cells group vs. $CD133^-$ cells group.

Figure S5. Correlation analysis of FXR or SOCS3 with markers of CSCs and stemness-related genes in HCC through Pearson analysis using TIMER database. (**A**) The correlation between NR1H4 and CSCs markers or stemness-related genes. (**B**) The correlation between SOCS3 and NR1H4. (**C**) The correlation between SOCS3 and CSCs markers or stemness-related genes.
