# Supplementary figures and images for: Farnesoid X Receptor Attenuates the Tumorigenicity of Liver Cancer Stem Cells by Inhibiting STAT3 Phosphorylation
Source: Int J Mol Sci. 2025 Jan 28;26(3):1122. doi: 10.3390/ijms26031122 (PMC11817294; doi:10.3390/ijms26031122)

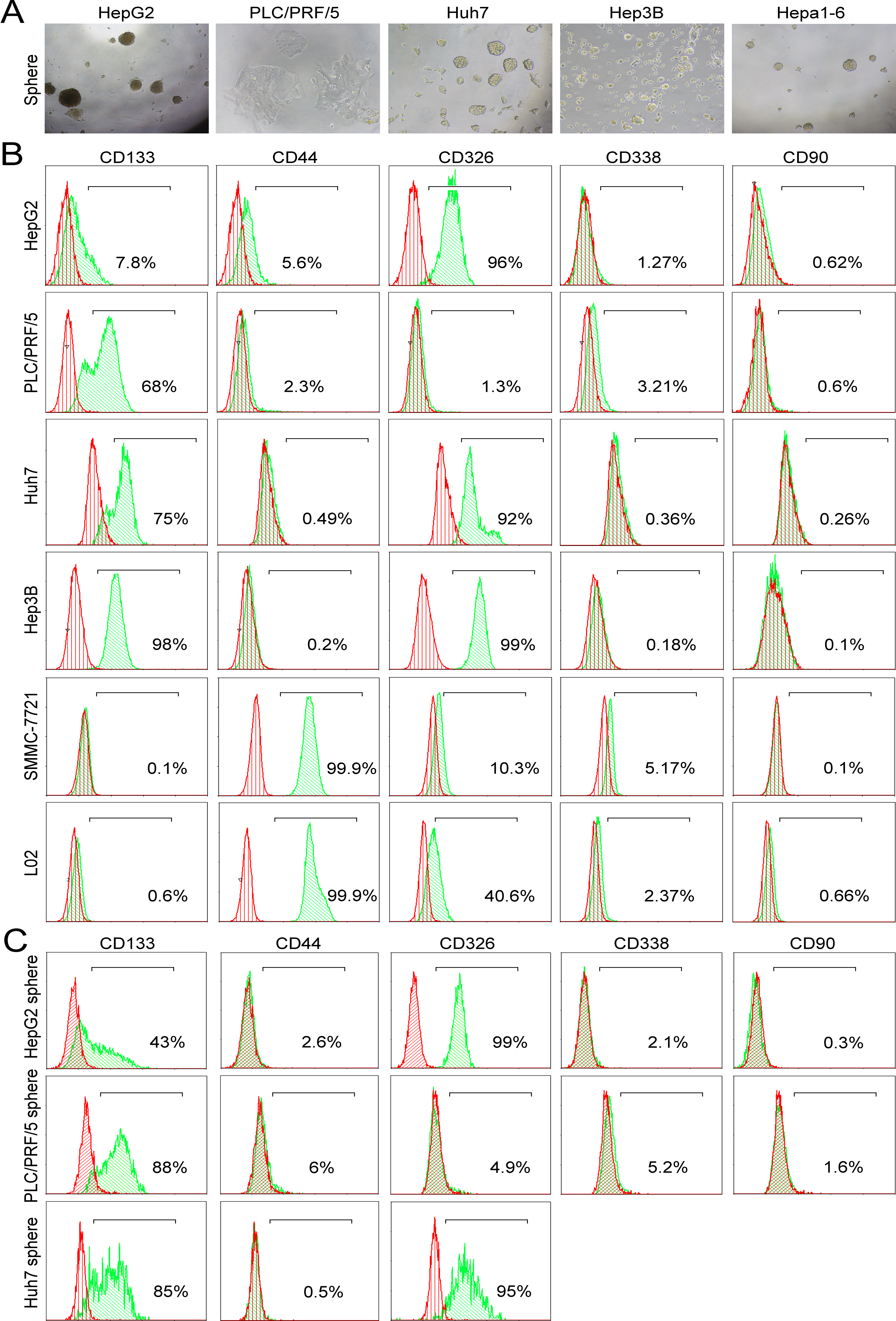

Supplement: Supplementary file 1 [file ijms-26-01122-s001.zip › Fig S1.tif]

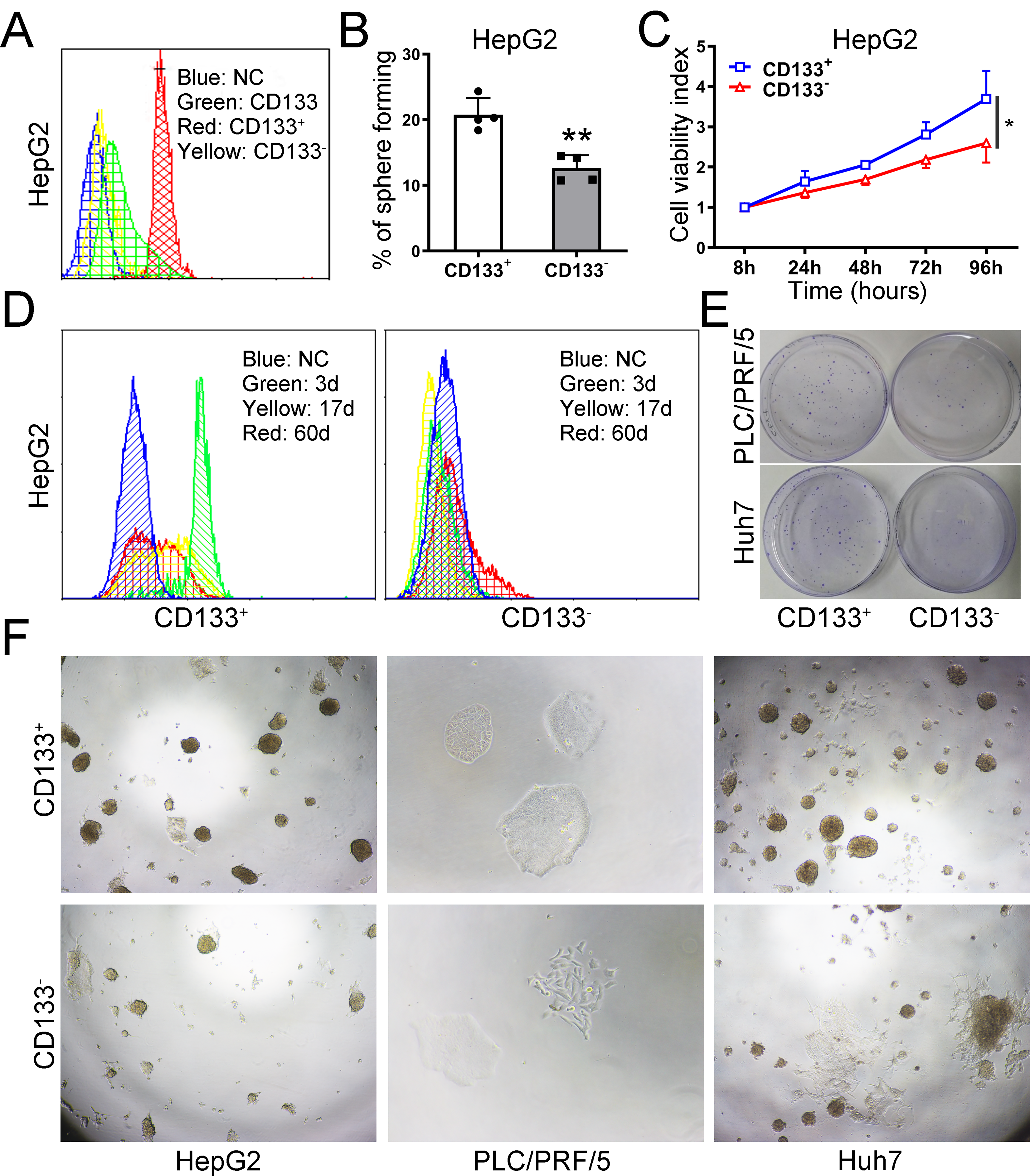

Supplement: Supplementary file 1 [file ijms-26-01122-s001.zip › Fig S2.tif]

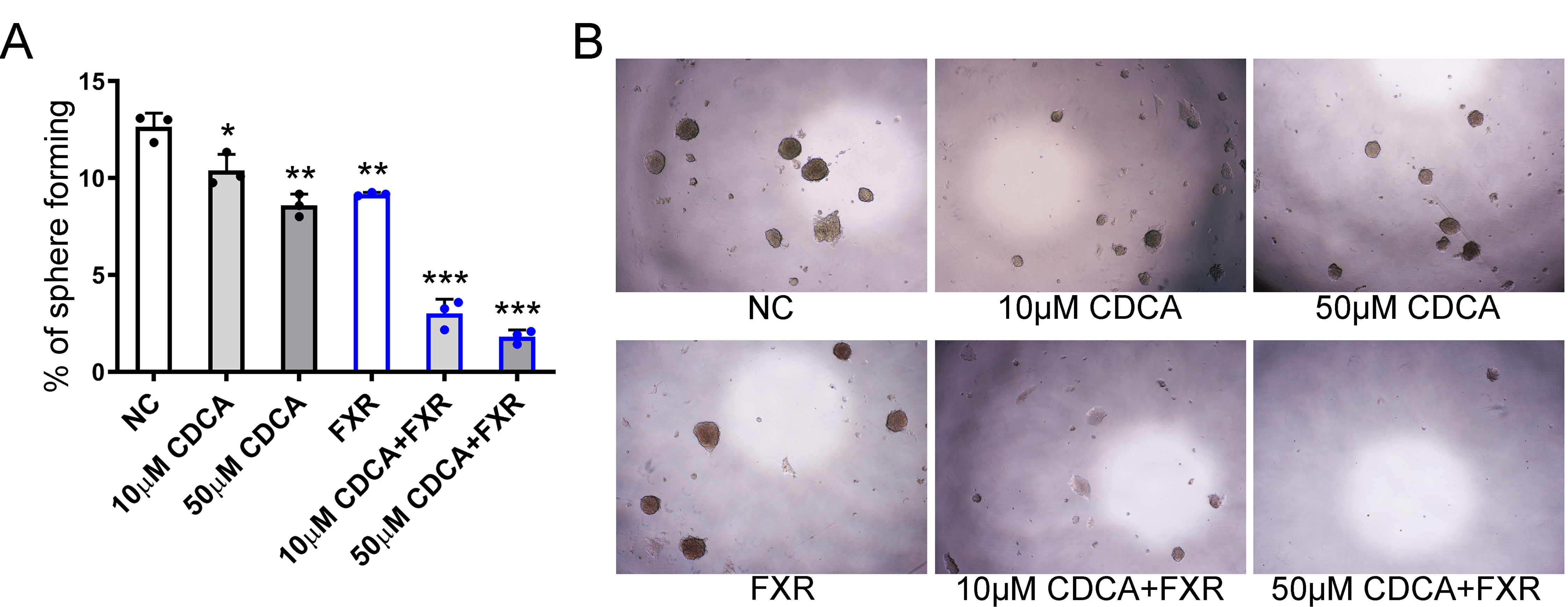

Supplement: Supplementary file 1 [file ijms-26-01122-s001.zip › Fig S3.tif]

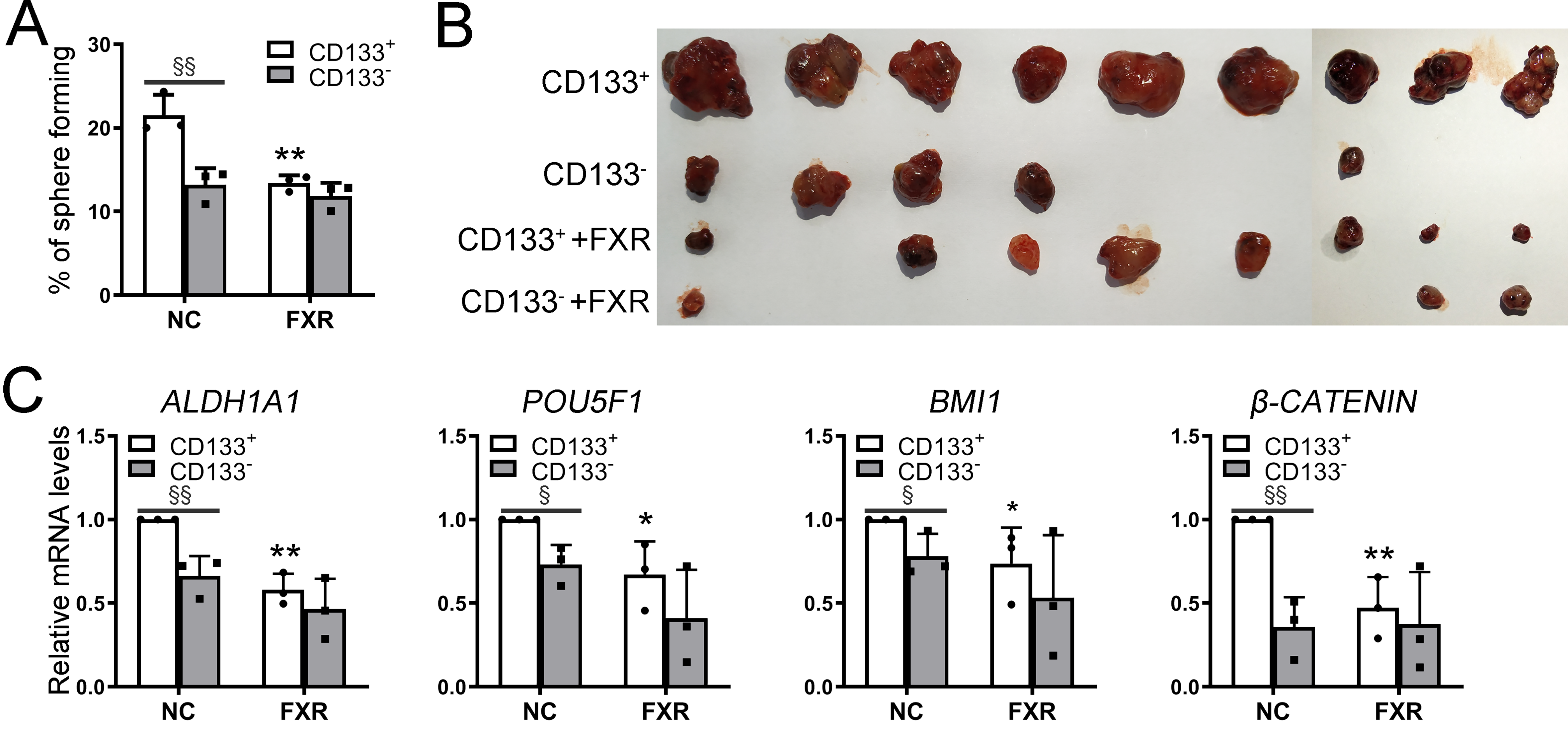

Supplement: Supplementary file 1 [file ijms-26-01122-s001.zip › Fig S4.tif]

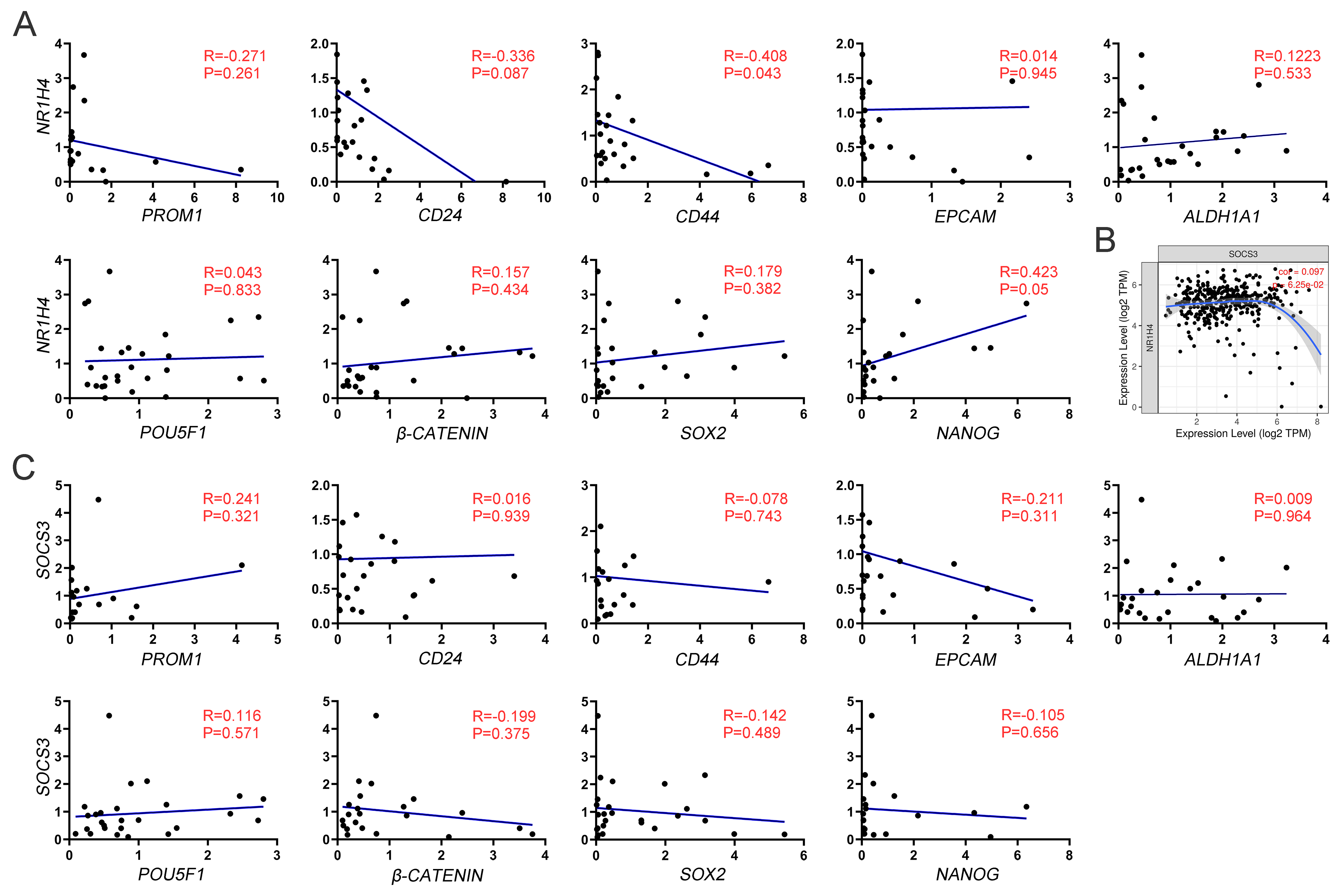

Supplement: Supplementary file 1 [file ijms-26-01122-s001.zip › Fig S5.tif]
